# Clinical significance of intramyocardial dissecting hematoma after MI: A review

**DOI:** 10.1097/MD.0000000000045650

**Published:** 2025-10-31

**Authors:** Qinqin Yu, Chang Zhou, Rong Liu, Ronghui Bao

**Affiliations:** a Department of Ultrasound, The First College of Clinical Medical Science, China Three Gorges University, Yichang, Hubei Province, China.

**Keywords:** diagnosis, intramyocardial dissecting hematoma, myocardial infarction, treatment

## Abstract

Intramyocardial dissecting hematoma (IDH) is a rare and catastrophic complication of myocardial infarction (MI) associated with high mortality and poor prognosis. Caused by hemorrhagic dissection between spiral myocardial fibers, IDH forms a blood-filled cavity that differs from complete cardiac rupture in that it preserves the integrity of the myocardial wall and is classified as a subtype of subacute cardiac rupture. Clinical manifestations vary according to the degree of impact on cardiac structure and hemodynamics. Echocardiography and cardiac magnetic resonance are essential in the diagnosis and assessment of IDH. Optimal management strategies for this rare complication of MI have not been established, and the ongoing debate about pharmacological versus surgical interventions requires multidisciplinary discussion to determine personalized treatment plans. Given the diagnostic and therapeutic challenges of IDH, there is an urgent need for clinicians to increase their understanding in order to improve diagnostic and therapeutic capabilities. This review aims to discuss the pathophysiological mechanisms, symptoms, diagnosis, treatment and prognosis strategies of IDH after MI.

## 1. Introduction

Intramyocardial dissecting hematoma (IDH), also known as intramural hematoma, intramural dissecting hematoma and intramyocardial dissection (ID), is a rare and potentially life-threatening complication that typically arises during myocardial infarction (MI) and its subsequent remodeling.^[1]^ Additionally, IDH can occur during percutaneous coronary intervention (PCI),^[2]^ coronary artery bypass grafting,^[3]^ radiofrequency ablation,^[4]^ severe chest trauma,^[5]^ or spontaneously.^[6]^ It has been described as a subacute incomplete cardiac rupture, characterized by hemorrhagic dissection of spiral myocardial fibers with preservation of endocardium and epicardium integrity, creating a neocavitation enclosed by myocardium.^[1]^ Although hemorrhagic dissection does not extend to the epicardium, it nevertheless increases the risk of death.^[7]^ IDH can occur in the left ventricular (LV) free wall, interventricular septum (IVS), or right ventricular (RV) free wall.^[8]^ It may extend,^[9]^ rupture into adjacent structures,^[10]^ and reabsorb spontaneously.^[11]^ The clinical presentation of IDH is variable, ranging from asymptomatic remission to cardiac death. Echocardiography and cardiac magnetic resonance (CMR) can confirm and assess the progressive nature of IDH.^[12]^ Management of these patients requires individual clinical judgement based on the choice of conservative treatment or surgical intervention.^[13]^ Due to the rarity and atypical clinical presentation of IDH after MI, it is poorly recognized by many clinicians, and is often misdiagnosed or overlooked at the time of initial diagnosis.^[14]^ It is therefore particularly important to increase clinicians’ awareness of IDH after MI. The aim of this review is to provide clinicians with a comprehensive cognitive framework to improve their diagnostic and therapeutic capabilities. To this end, the existing studies on IDH after MI are reviewed and the pathophysiological mechanisms, symptoms, diagnosis, treatment and prognosis are discussed.

## 2. Methods

For this study, we conducted a comprehensive search of the English-language medical literature in the PubMed, identifying articles related to IDH following MI. The search covered the period from the inception of the database to October 2024. We used combinations of the following keywords to search for published studies: “MI,” “IDH,” “intramural hematoma,” “intramural dissecting hematoma,” “ID,” “epidemiology,” “diagnosis,” “treatment” and “prognosis.” The inclusion criteria were restricted to studies involving patients with IDH after MI, including original research articles, case reports and review articles. We excluded duplicate publications, studies unrelated to our research topic, articles not accessible in full text, conference abstracts, and editorials. Additionally, we manually screened the search results to ensure the accuracy and completeness of the included literature. The ethics committee of Yichang Central People’s Hospital approved the study (approval number: 2025-233-01).

## 3. Pathophysiological mechanisms

IDH represents a rare and catastrophic complication associated with MI. The pathophysiological mechanism is a complex and multifactorial process. The underlying mechanism involves hemorrhagic dissection between myocardial fibers, which is typically triggered by increased vascular fragility in the MI area, diminished tissue tensile strength, or a sudden increase in coronary capillary perfusion pressure.^[10,15]^ While reperfusion therapy can salvage some myocardium, it may also lead to reperfusion injury. The compromised microvasculature may rupture under pressure, resulting in the formation of IDH.^[16]^ Long-term chronic ischemia promotes structural and functional remodeling of the microvasculature, altering their biomechanical properties and increasing the susceptibility of these vessels to rupture and hemorrhage during reperfusion.^[16]^ Liu et al^[17]^ study further elucidates this process, where even after thrombolysis for culprit vessels in patients with MI achieving thrombolysis in MI 3 flow, 80% of patients may still experience microvascular obstruction and bleeding, leading to IDH. Nevertheless, early revascularisation, particularly within 72 hours of the onset of MI, has the potential to prevent or reduce the risk of reperfusion-related hemorrhage and may facilitate the salvage of myocardium. Conversely, delayed medical attention may increase the risk of IDH following MI.^[7]^ Furthermore, the risk of IDH may be intensified by elevated endothelial cell pressure, inflammatory responses, and disruption of endothelial cell junctions following MI.^[18]^ The spiral arrangement of the myocardium provides the anatomical basis for myocardial dissection, whereby the diffusion of blood along the spiral myofibres is promoted.^[19]^ Conversely, IDH may represent a potential mechanism underlying LV remodeling and heart failure following MI.^[20]^

## 4. Dissection site and MI location

Pliam et al^[21]^ conducted a pioneering study in 1993, in which they retrospectively analyzed 15 cases of coronary artery disease and recent MI complicated by IDH and found that the IVS was the most commonly affected site, including 7 cases of inferior MI and 1 case of anterior MI. Subsequently, in 2009, Vargas-Barron et al,^[22]^ using intraoperative or necropsy studies of 15 patients with acute MI (AMI), came to similar conclusions, with the IVS (9 cases) being the most commonly involved site, followed by the LV free wall (6 cases). However, in their study, 13 cases were anterior MI and 2 were inferior MI.^[22]^ Zhao et al^[23]^ in 2016 performed a more comprehensive literature review of 68 patients with IDH after AMI, including previously reported cases,^[21,22]^ and found that IDH predominantly involved the LV free wall (47%), followed by the IVS (26.5%) and the RV free wall (26.5%). In 32 patients with LV free wall dissection, 53% of MI locations were in the apex, anterior, anteroseptal, or anterolateral wall, while 47% were in the inferior, posterior, or lateral wall.^[23]^ In 18 patients with septal dissection, 66.7% of MI locations were in the anteroseptal or apical segment, and 33.3% were in the posteroseptal or inferior wall.^[23]^ Similarly, in 18 patients with RV free wall dissection, 94.4% were in the inferior or posterior wall.^[23]^ Through a meta-analysis of the literature, Leitman et al^[24]^ in 2018 found that 66% of IDH after MI cases were associated with anterior MI. A systematic review by Hajsadeghi et al^[25]^ in 2020 examined 37 cases of IDH with ventricular septal rupture (VSR) following MI and found that the IVS was the most commonly involved site (56.8%), followed by the RV (24.3%), both IVS and RV (13.5%), and the LV apex (5.4%), which were relatively rare. Anterior and inferior MI were observed in 14 and 23 cases, respectively.^[25]^ These studies documented that IDH typically forms in the myocardium adjacent to the culprit coronary artery lesion. The location of the MI is mainly the anterior wall and inferior wall. The dissection site of IDH following MI is primarily focused on the LV free wall, the IVS and the RV free wall, with isolated occurrence in the atria being very rare.^[25]^ Kovacic et al^[26]^ described a case in which a distal right coronary artery occlusion led to myocardial dissection from the inferior MI area, crossing the atrioventricular groove and forming IDH in the left atrium. In addition, IDH may extend into adjacent structures or rupture. Wilson et al^[9]^ reported a case of LV IDH (LVIDH) occurring after MI and extending into the left atrial wall. We reported an unusual case of post-MI LVIDH penetrated the RV outflow tract (LVOT) resulting in LVOT obstruction.^[27]^

## 5. Symptoms and Electrocardiogram changes

### 5.1. Symptoms

Zavar et al^[28]^ conducted a retrospective study showing that over 75% of patients with IDH had a primary etiology of MI, typically occurring within 7 to 10 days of MI. Notably, even in cases of old MI, the progression to IDH is possible.^[28]^ In the largest published series of patients with IDH following AMI, the mean age was 66 ± 10 years, and the majority were male with a history of hypertension and diabetes.^[23]^ The clinical manifestations of IDH after AMI are diverse, depending primarily on the location, size of the hematoma, communication with the ventricles, effect on valvular function and whether the outflow tract is obstructed. Because of the varying degrees of impact on cardiac structure and hemodynamics, the clinical symptoms of patients with IDH is also variable.

Stöllberger et al^[29]^ reported the case of a 68 years old man who developed atrial fibrillation and third-degree atrioventricular block after subacute posterior MI, followed by ventricular fibrillation on the 4th day of hospitalization. Initially misdiagnosed as isolated abnormal trabeculations in the LV lateral wall, the patient was later surgically confirmed to have lateral wall IDH. Meyers et al^[30]^ described a 68-year-old diabetic male patient with recurrent chest pain, ST-segment elevation, mild pulmonary edema and AF whose LVIDH associated with inferior MI masqueraded as a pseudoaneurysm. Cicenia et al^[20]^ reported a 72-year-old male patient undergoing PCI for AMI who, despite being asymptomatic, was found to have LVIDH on echocardiography prior to discharge. These cases illustrate the diverse clinical symptoms of IDH following MI, which lacks typical clinical features and is prone to misdiagnosis or missed diagnosis, requiring thorough diagnostic investigations to confirm the correct etiology.

### 5.2. ECG changes

The ECG plays an important role in the diagnosis of IDH after MI. In more than half of IDH cases, bundle branch block can be detected on ECG.^[31]^ In addition, patients may present with persistent ST-segment elevation beyond 72 hours after initial presentation.^[12]^ In patients with AMI, ECG evidence of persistent ST-segment elevation in relevant leads or new ST-segment elevation (excluding recurrent infarction) should prompt clinicians to consider the possibility of IDH as a complication. Therefore, timely and frequent ECG reevaluation is essential for the diagnosis of IDH. Changes in the ECG not only reflect the presence of IDH, but may also indicate its effect on myocardial perfusion. If IDH enlarges and compresses the coronary arteries, the ECG may show signs of myocardial ischemia or even changes characteristic of MI. Ventricular arrhythmias are common in patients at risk of sudden cardiac death and should be monitored closely.^[31]^ Galiuto et al^[32]^ reported a case of spontaneous IDH in a patient with antiphospholipid antibody syndrome whose ECG presentation closely mimicked inferior MI, adding complexity to the diagnosis of IDH but also highlighting the important role of the ECG in identifying such special cases. In clinical practice, we should pay close attention to the dynamic changes in the ECG and combine them with the patient’s history, symptoms, physical signs and necessary imaging studies to make an accurate diagnosis and differential diagnosis.

## 6. Diagnosis

IDH is a neocavitation entirely contained within the myocardium, resulting from hemorrhagic dissection between spirally arranged myocardial fibers,^[1]^ which may expand,^[9]^ rupture into adjacent structures^[10]^ or resolve spontaneously.^[11]^ The diagnosis of IDH can be made at different clinical and evolutionary stages, ranging from early IDH to severe pseudoaneurysm formation.^[33]^ In the early stages, IDH presents as a “closed ID” with no communication with the LV cavity.^[33]^ In severe or advanced stages, the ID may tear into the LV cavity, resulting in a false-pseudoaneurysm or “open ID.”^[33]^ There are various terminologies used in the literature, including “pseudo-falseaneurysm” or “pseudo-pseudoaneurysm,”^[34,35]^ which may lead to confusion. Carrión-Sánchez et al^[33]^ propose “open ID” as a unique and more comprehensive term to standardize the terminology of IDH. They believe that this term not only meets the requirements of multimodality imaging modalities, but is also crucial to ensure accurate differential diagnosis and selection of personalized management.^[33]^ Early and accurate diagnosis of IDH is challenging; prior to 1980, IDH was primarily diagnosed by autopsy and surgery,^[36]^ but newer multimodality imaging techniques may help to confirm and assess the progressive nature of IDH.

### 6.1. Echocardiography

Since Hodsden et al^[36]^ reported the first case of IDH diagnosed by echocardiography in 1981, the development of high-resolution echocardiographic technology has made it possible to diagnose IDH promptly and accurately. For the majority of patients who are hemodynamically unstable and unable to undergo CT or CMR, transthoracic echocardiography (TTE) has become the preferred noninvasive technique for the diagnosis of IDH. Continuous echocardiography helps to determine the evolution of IDH and can provide critical evidence to guide surgical outcomes and the need for treatment.^[9]^ Colour Doppler ultrasound can detect communication with the endocardium or pericardial cavity.^[10]^ Vargas-Barón et al^[22]^ proposed that IDH can be diagnosed if a patient’s echocardiogram shows at least 3 of the following characteristics: 1 or more newly formed cavities with echo-lucent centers; thin and mobile endocardium surrounding the cavity; a cystic area surrounded by normal myocardial tissue; partial or complete disappearance of the cystic cavity; echo changes suggesting the presence of blood; communication between the cavity and the ventricles; detection of color Doppler flow within the cavity. Lee et al^[37]^ used continuous TTE to identify IDH that appeared in late anterior MI and mimicked LV thrombus on imaging. Continuous TTE helped to assess and differentiate between these 2 pathologies, sparing patients unnecessary anticoagulation therapy. Carcagní et al^[38]^ failed to diagnose IDH on the first TTE, but the second TTE with contrast-enhanced ultrasound (CEU) revealed an anechoic cavity within the myocardium and bidirectional flow between this cavity and the LV, which was initially misdiagnosed as a LV pseudoaneurysm. It was not until the third TTE, when the anechoic cavity was observed to extend into the myocardial wall, that the correct diagnosis of IDH was made.^[38]^ TTE, with its convenience, noninvasiveness and bedside availability, can accurately assess disease progression and allow precise treatment decisions for patients.^[39]^

The differential diagnosis of IDH includes prominent ventricular trabeculations, intracavitary thrombi and pseudoaneurysms. Careful examination of the endocardial and epicardial layers surrounding IDH is essential. CEU can help to define endocardial borders, continuity of prominent myocardial echoes, whether there is contrast penetration between the myocardium and ventricles, and whether there is contrast enhancement in the pericardial space, which has greater diagnostic value than conventional echocardiography.^[40]^ IDH shows variable degrees of enhancement on contrast-enhanced imaging due to active bleeding and hematoma formation.^[41]^ Therefore, the degree of enhancement of the hematoma can inform the clinician whether there is ongoing bleeding. The combination of echocardiography and CEU is noninvasive, safe, economical, can be performed at the bedside, and accurately indicates the location and size of IDH, the presence of shunts, and allows real-time observation of hemodynamic changes and myocardial perfusion.^[42]^

Although TTE is considered an important initial diagnostic tool for IDH, its diagnostic results are often equivocal due to the influence of acoustic window conditions such as the chest wall and pulmonary air.^[43]^ This can make it difficult to distinguish IDH from LV thrombus, pseudoaneurysm or trabeculations, increasing the risk of missed or incorrect diagnoses. Compared to TTE, transesophageal echocardiography (TEE) offers higher image resolution and clearer details of cardiac structures. Vargas-Barón et al^[44]^ emphasized the importance of 2D-TEE in the diagnosis and management of IDH after MI, noting that 3D TEE can provide 3-dimensional and comprehensive information about cardiac structures, helping clinician to accurately determine the spatial location and morphology of IDH, thereby effectively differentiating IDH from intracardiac thrombus and pseudoaneurysm, enhancing diagnostic precision.

### 6.2. CMR

Echocardiography is a diagnostic imaging modality that relies on the diagnostic skills of the sonographer and the image quality of the patient, and if it is difficult to confirm the integrity of the epicardium, IDH may be missed or misdiagnosed as a LV thrombus or pseudoaneurysm.^[39]^ CMR can provide more accurate structural images of IDH, complementing the diagnosis made by echocardiography,^[12,39]^ and is therefore recommended in clinically stable patients.^[10]^ CMR is the gold standard for the diagnosis of IDH,^[24]^ with its major advantage being its high contrast resolution, which can accurately differentiate IDH from normal myocardial tissue, and it has additional advantages in detecting microvascular obstruction (MVO) and late myocardial enhancement.^[43]^ The cine steady-state free precession sequence in CMR can provide excellent visualization of the LV, the typical anatomical structures of IDH, dissecting endocardial flap, and communication with the RV.^[18]^ T1 and T2 sequences are highly sensitive to blood products and are commonly used to aid in the diagnosis of IDH.^.[45]^ T1-weighted imaging may show high signal lesions in the affected areas, which may be clinically interpreted as blood products associated with subacute hemorrhage.^[45]^ T2-weighted imaging may show high signal foci corresponding to fat or edema.^[45]^ Fat suppression techniques help to differentiate between edema and fat.^[18]^ Delayed enhancement images may show a ring of high signal infarcted myocardium and the typical flap-like endocardial tear surrounding IDH.^[18]^ The combination of late gadolinium enhancement (LGE), T2-weighted imaging, resting perfusion and early gadolinium enhancement can help to differentiate IDH from intracavitary thrombi.^[46]^ CMR is a powerful investigative tool that can reveal the potential interactions of intramyocardial hemorrhage and thus plays a key role in ischemia-reperfusion injury.^[18]^

**Figure 1.**
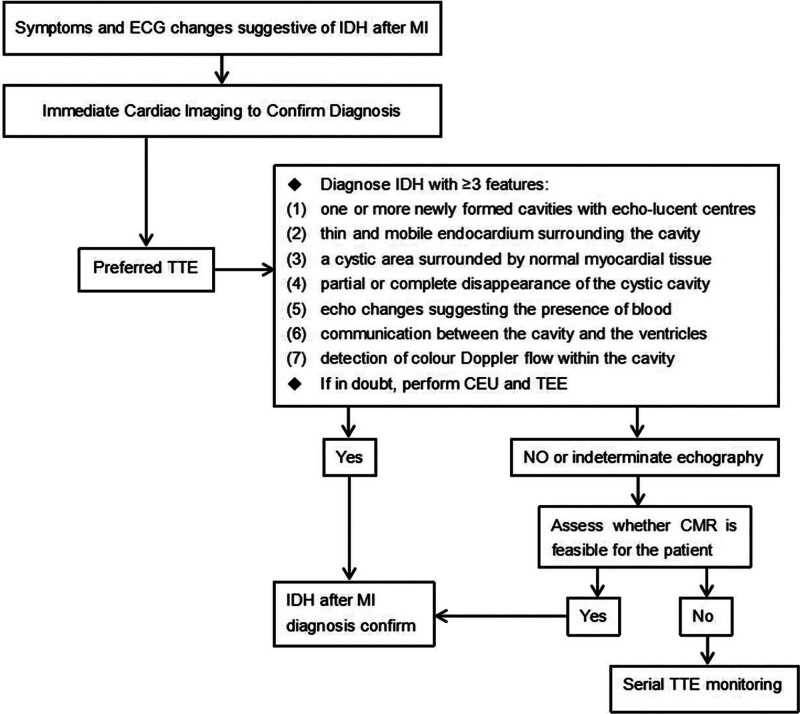
Diagnostic flowchart for IDH after MI. CEU = contrast-enhanced ultrasound, CMR = cardiac magnetic resonance, ECG = electrocardiogram, IDH = intramyocardial dissecting hematoma, MI = myocardial infarction, TEE = transesophageal echocardiography, TTE = transthoracic echocardiography.

However, the optimal choice of CMR sequence for IDH detection remains controversial. Kumar et al^[47]^ suggested that T2* CMR sequences can effectively quantify myocardial reperfusion hemorrhage. Furthermore, Kali et al^[48]^ suggested that T2* is more suitable than T2-weighted CMR for the detection of acute IDH. The study by Arai et al^[49]^ confirmed the accuracy of T1-CMR in measuring the volume of IDH. Sakrana et al^[45]^ found that IDH was clearly demonstrated on cine steady-state precession sequences (FIESTA), delayed inversion recovery sequences (DIR) (both T1- and T2-weighted), but on LGE imaging, the enhanced infarcted endocardial layer was difficult to distinguish from the adjacent bright LV blood. Therefore, they recommended the use of dark-blood LGE techniques to counteract the magnetization effect of LV blood for better detection of ischemic myocardial scar.^[45]^ These studies indicate that CMR has significant value in the diagnosis and assessment of IDH. Although CMR is a highly accurate imaging modality, there are certain limitations to its use, such as long examination times, high cost, not being the first choice examination modality in critically ill patients, and scanning issues in patients with implanted pacemakers and cardiac devices. A diagnostic flowchart for IDH after MI is shown in Figure 1.

## 7. Treatment and Prognosis

IDH is a serious complication of MI with an extremely high mortality rate and a generally poor prognosis. There is limited evidence regarding the treatment and prognosis of IDH after MI, and most clinicians’ current knowledge is based on case reports.^[14]^ In 1993, Pliam et al^[21]^ first retrospectively analyzed 15 cases of IDH after coronary artery disease and recent MI, in which 10 patients received medical treatment with only 1 survivor, while 5 patients underwent surgical treatment and all survived. In 2009, Vargas-Barrón et al^[22]^ reported 15 cases of IDH after MI, of which 5 were treated medically with a mortality rate of 20%, 8 were treated surgically with a mortality rate of 80% and 2 underwent PCI with a mortality rate of 50%. Furthermore, Vargas-Barrón et al^[22]^ found that 9 patients with interventricular septal hematoma (IVSH) had an extremely poor prognosis with a mortality rate of up to 78%, whereas patients with free wall IDH had a survival rate of 100% after 12 months of follow-up. In 2016, Zhao et al^[23]^ analyzed 68 cases of MI after IDH in the literature regarding the location of the hematoma and the efficacy of medical and surgical treatment. They found no difference in survival between medical and surgical treatment regardless of the location of the IDH in the LV, but patients with RV free wall IDH significantly benefited from surgical repair. In 2020, Hajsadeghi et al^[25]^ reviewed 37 cases of IDH after MI complicated by VSR, and the results showed that the mortality rate was higher in patients treated conservatively than in those treated surgically. These retrospective analyses indicate that the mortality rate of IDH after MI is high with both conservative and surgical treatment, and there is controversy regarding the optimal treatment plan.

Leitman et al^[24]^ conducted a meta-analysis of mortality prediction factors in 40 patients with IDH and found that LV ejection fraction (LVEF) <35%, age >60 years and longer time to definitive diagnosis were predictive of in-hospital mortality, with LVEF <35% being the strongest independent predictor of mortality. Spinelli et al^[50]^ studied 87 cases of anterior ST-segment elevation MI (STEMI), 42 of which were associated with IDH, and found that IDH was correlated with adverse angiographic outcomes, severe impairment of LV function, adverse LV remodeling and poorer long-term prognosis. Zavar et al^[28]^ studied 77 cases of IDH and found that patients had a 3.92-fold increased risk of death due to pericardial effusion and that VSR may increase the risk of adverse outcomes by 1.33-fold. Therefore, early diagnosis and intervention for IDH is of paramount importance.

Optimal management guidelines for IDH after MI are not yet standardized. The choice between conservative management and surgical intervention is influenced by a variety of factors, including the patient’s hemodynamic stability, accurate and timely assessment of the IDH structure, LV function, presence of complications, and the clinical experience of each surgical center.^[48]^ Patients with apex-localized IDH and hemodynamic stability, especially those with a previously revascularised culprit artery, tend to have spontaneous resolution of the hematoma and can be managed conservatively.^[24]^ In addition, conservative management is a viable option for hemodynamically stable patients with large IDH or impaired LV function^.[45]^ Patients with IDH following anterior MI who are hemodynamically stable without dissecting endocardial flap or pseudoaneurysm may achieve early spontaneous resolution of IDH with conservative management.^[11]^ If IDH presents with thrombus formation at the entry tear, early discontinuation of anticoagulation, adjustment of antiplatelet agents and a relatively conservative conservative approach may be adopted, as this thrombus may seal the IDH cavity, reduce pressure on the damaged myocardial wall, reduce the risk of rupture and promote blood stasis, facilitating complete thrombosis of the IDH.^[51]^ In cases where the risk of major surgical complications and hemodynamic stability appear to allow conservative management, good medium-term results can be achieved.^.[52]^ However, in the presence of hemodynamic instability or an expanding hematoma with pericardial effusion, urgent surgical repair should be considered.^[52]^ Patients with a low LVEF, hemodynamic compromise, VSR, and continuous echocardiographic evidence of expanding IDH, especially those with anterior MI, should undergo surgical treatment.^[28]^ Reports of surgical treatment include CABG, median sternotomy, hematoma evacuation and subsequent ventricular wall repair; however, these surgical treatments are performed in patients without cardiogenic shock.^[53]^ Early (<7 days) repair of VSR is associated with a high mortality rate, and severely reduced LV function, large IVSH with extensive MVO and ventricular arrhythmias, heart transplantation is considered the safest option.^[43]^ Due to the rarity of IDH after MI and the lack of supporting evidence, there remains a controversy between conservative and surgical management, and current guidelines for acute coronary syndrome do not mention or specify this potential complication.^[54]^ Therefore, more evidence is needed to determine the best management approach. At present, the management of IDH should involve a multidisciplinary discussion with cardiothoracic surgeons and a cardiology team, including heart failure specialists and interventional cardiologists, to decide on a personalized treatment plan for the patient.

## Author contributions

**Investigation:** Qinqin Yu, Rong Liu.

**Methodology:** Qinqin Yu, Chang Zhou.

**Writing – original draft:** Qinqin Yu, Chang Zhou.

**Writing – review & editing:** Qinqin Yu, Chang Zhou, Rong Liu, Ronghui Bao.
